# 8-Phenyl-16-thia­penta­cyclo­[6.6.5.0^1,18^.0^2,7^.0^9,14^]nona­deca-2,4,6,9,11,13,18-hepta­ene

**DOI:** 10.1107/S1600536813017285

**Published:** 2013-06-29

**Authors:** Eason M. Mathew, M. Sithambaresan, P. A. Unnikrishnan, M. R. Prathapachandra Kurup

**Affiliations:** a Department of Applied Chemistry, Cochin University of Science and Technology, Kochi 682 022, India; b Department of Chemistry, Faculty of Science, Eastern University, Sri Lanka, Chenkalady, Sri Lanka

## Abstract

In the title compound, C_24_H_18_S, the dihedral angles between the phenyl ring and the two benzene rings of the anthracene moiety are 51.92 (9) and 68.24 (9)°, whereas the dihedral angle between the two anthracene benzene rings is 120.13 (9)°. The three non-aromatic six-membered rings are in boat conformations, while the five-membered ring has an envelope conformation on the S atom. In the crystal, there are three C—H⋯π inter­actions, which facilitate the packing of the mol­ecules.

## Related literature

For background to dibenzobarrelene dervatives and their applications, see: Khalil *et al.* (2010 ▶); Cox *et al.* (2013 ▶). For the synthesis of related compounds, see: Ciganek (1980 ▶); Vetter (1998 ▶). For puckering parameters, see: Cremer & Pople (1975 ▶).
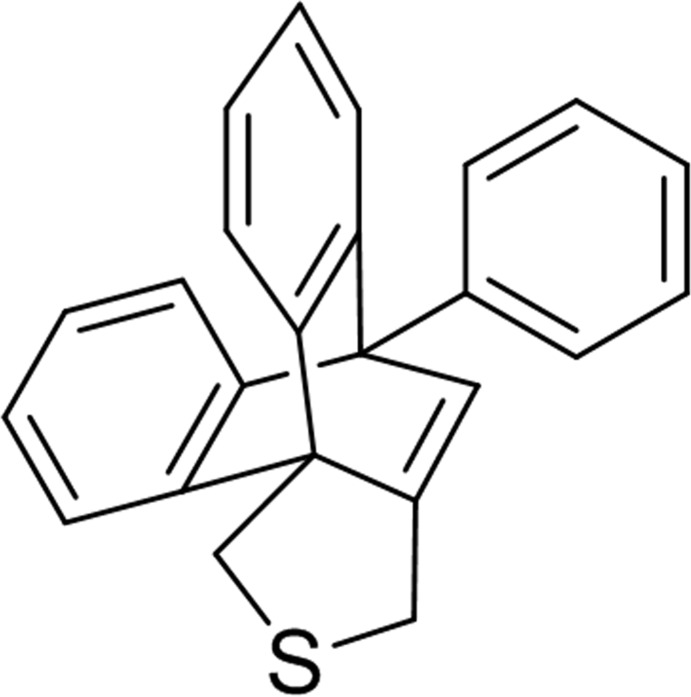


## Experimental

### 

#### Crystal data

C_24_H_18_S
*M* *_r_* = 338.44
Orthorhombic, 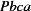

*a* = 18.8842 (11) Å
*b* = 9.5339 (4) Å
*c* = 19.1140 (9) Å
*V* = 3441.3 (3) Å^3^
*Z* = 8
Mo *K*α radiation
μ = 0.19 mm^−1^
*T* = 296 K
0.30 × 0.25 × 0.20 mm

#### Data collection

Bruker Kappa APEXII CCD diffractometer
Absorption correction: multi-scan (*SADABS*; Bruker, 2004 ▶) *T*_min_ = 0.945, *T*_max_ = 0.963
22858 measured reflections
3757 independent reflections
2765 reflections with *I* > 2σ(*I*)
*R*_int_ = 0.032

#### Refinement

*R*[*F* ^2^ > 2σ(*F* ^2^)] = 0.042
*wR*(*F* ^2^) = 0.138
*S* = 1.02
3757 reflections
226 parameters
H-atom parameters constrained
Δρ_max_ = 0.18 e Å^−3^
Δρ_min_ = −0.26 e Å^−3^

### 

Data collection: *APEX2* (Bruker, 2004 ▶); cell refinement: *APEX2* and *SAINT* (Bruker, 2004 ▶); data reduction: *SAINT* and *XPREP* (Bruker, 2004 ▶); program(s) used to solve structure: *SHELXS97* (Sheldrick, 2008 ▶); program(s) used to refine structure: *SHELXL97* (Sheldrick, 2008 ▶); molecular graphics: *ORTEP-3 for Windows* (Farrugia, 2012 ▶) and *DIAMOND* (Brandenburg, 2010 ▶); software used to prepare material for publication: *SHELXL97* and *publCIF* (Westrip, 2010 ▶).

## Supplementary Material

Crystal structure: contains datablock(s) I, global. DOI: 10.1107/S1600536813017285/zl2554sup1.cif

Structure factors: contains datablock(s) I. DOI: 10.1107/S1600536813017285/zl2554Isup2.hkl

Supplementary material file. DOI: 10.1107/S1600536813017285/zl2554Isup3.cml

Additional supplementary materials:  crystallographic information; 3D view; checkCIF report

## Figures and Tables

**Table 1:** Hydrogen-bond geometry (Å, °) *Cg*1 and *Cg*2 are the centroids of the C19–C2 and C8–C13 rings, respectively.

*D*—H⋯*A*	*D*—H	H⋯*A*	*D*⋯*A*	*D*—H⋯*A*
C2—H2⋯*Cg*1^i^	0.93	2.80	3.516 (2)	135
C5—H5⋯*Cg*1^ii^	0.93	2.76	3.5844 (19)	149
C15—H15*B*⋯*Cg*2^iii^	0.97	2.98	3.845 (2)	149

Symmetry codes: (i) 
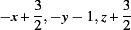
; (ii) 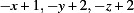; (iii) 
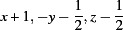
.

**Fig. 1.**
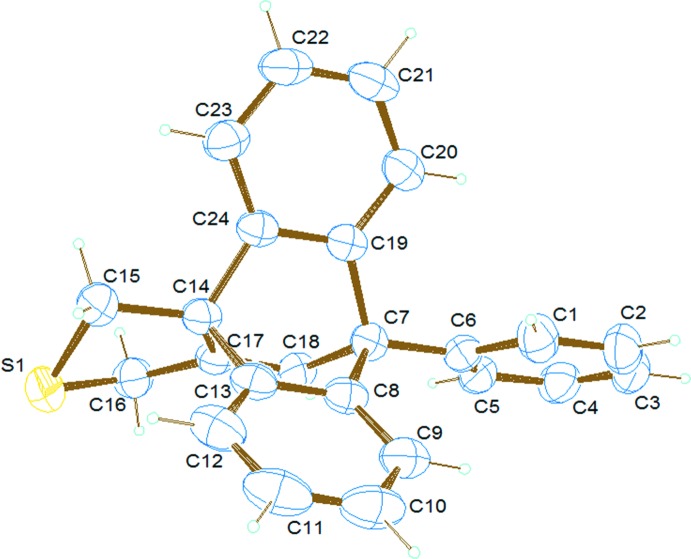
ORTEP view of the title compound drawn with 50% probability displacement ellipsoids for the non-H atoms.

**Fig. 2.**
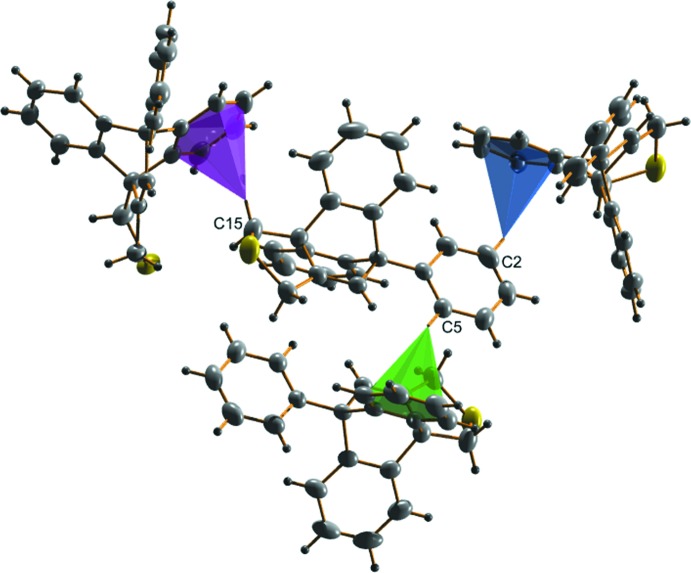
C—H···π interactions found in the title compound.

**Fig. 3.**
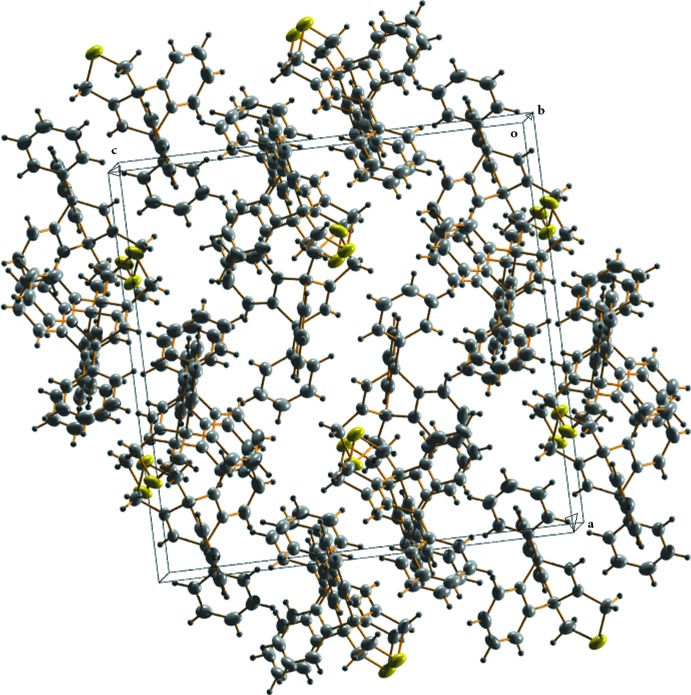
Packing diagram of the compound along b axis.

## supplementary crystallographic information

### Comment

Thiazoles, thiophenes and their derivatives have attracted continuing interest over the years since they often exhibit various biological activities and have been investigated for the treatment of various diseases (Khalil *et al.*, 2010). Dibenzobarrelene derivatives find application in the alignment of nematic liquid crystals. The strong coupling of the liquid crystal directors to dibenzobarrelene groups is responsible for the alignment mechanism (Cox *et al.*, 2013).

The compound (Fig. 1) crystallizes in the monoclinic space group *Pbca*. The dihedral angles between the phenyl ring and the other two benzene rings of the anthracene moiety are 51.92 (9) and 68.24 (9)°. The two aromatic rings of the anthracene moiety form a dihedral angle of 120.13 (9)° between themselves.

The five-membered heterocyclic ring C14–17/S1 is in an envelope conformation on S1 [φ = 3.9 (3)°] (Cremer & Pople, 1975). The ring C7/C8/C13/C14/C19/C24 of the three fused six-membered rings is in a boat conformation [φ = 180.31 (13)° and θ = 89.60 (12)°] with a total puckering amplitude Q_T_ of 0.8317 (18) Å. The second six-membered ring C7/C8/C13/C14/C17/C18 is also in a boat conformation [φ = 359.90 (13)° and θ = 89.16 (13)°] having a total puckering amplitude Q_T_ of 0.8160 (18) Å. The third six-membered ring C7/C14/C17/C18/C19/C24 also has the same conformation [φ = 358.83 (13)° and θ = 89.80 (13)°] and has a total puckering amplitude Q_T_ of 0.7856 (18) Å.

There are three weak C–H···π interactions (Fig. 2) between the H atoms attached at the C2, C5 and C15 atoms and neighboring aromatic rings. The hydrogen atoms from the C2 and C5 atoms form C–H···π interactions with the C19—C24 ring of two adjacent molecules from opposite sides of the main molecule and the hydrogen attached at C15 atom has an interaction with the C8—C13 ring of a neighbouring molecule with H···π distances of 2.80, 2.76 and 2.98 Å, respectively (Table 1). The packing of molecules is dominated by these C–H···π interactions. Fig. 3 shows the packing diagram of the title compound along *b* axis.

### Experimental

The title compound was prepared by adapting a reported procedure (Ciganek, 1980; Vetter, 1998). 10-phenyl-9-anthracenemethanol (1 mmol) obtained by reduction of the corresponding aldehyde (1 mmol) using sodium borohydride (3 mmol) and thiourea (2 mmol) were dissolved in acetone and stirred overnight with 10 ml of 5 N HCl to obtain10-phenyl-9-anthracenemethanethiol which was converted into 10-phenyl-9-anthracenemethyl propargyl sulfide through treatment with propargyl bromide. 10-Phenyl-9-anthracenemethyl propargyl sulfide was refluxed in *p*-xylene for 7 h (intramolecular Diels-Alder reaction) to get the title compound. Colourless crystals suitable for X-ray structure determination were recrystallized from acetonitrile by slow evaporation over a few days (m.p: 218 °C).

### Refinement

All H atoms on C were placed in calculated positions, guided by difference maps, with C–H bond distances of 0.93–0.97 Å. H atoms were assigned *U*_iso_=1.2U_eq_. Omitted owing to bad disagreement were the reflections (0 0 2), (2 0 0), and (1 0 2).

### Crystal data

**Table:**

C_24_H_18_S	*F*(000) = 1424
*M**_r_* = 338.44	*D*_x_ = 1.306 Mg m^−^^3^
Orthorhombic, *P**b**c**a*	Mo *K*α radiation, λ = 0.71073 Å
Hall symbol: -P 2ac 2ab	Cell parameters from 5274 reflections
*a* = 18.8842 (11) Å	θ = 2.4–27.6°
*b* = 9.5339 (4) Å	µ = 0.19 mm^−^^1^
*c* = 19.1140 (9) Å	*T* = 296 K
*V* = 3441.3 (3) Å^3^	Block, colorless
*Z* = 8	0.30 × 0.25 × 0.20 mm

### Data collection

**Table:**

Bruker Kappa APEXII CCD diffractometer	3757 independent reflections
Radiation source: fine-focus sealed tube	2765 reflections with *I* > 2σ(*I*)
Graphite monochromator	*R*_int_ = 0.032
Detector resolution: 8.33 pixels mm^-1^	θ_max_ = 27.0°, θ_min_ = 2.6°
ω and φ scan	*h* = −24→20
Absorption correction: multi-scan (*SADABS*; Bruker, 2004)	*k* = −11→12
*T*_min_ = 0.945, *T*_max_ = 0.963	*l* = −24→24
22858 measured reflections	

### Refinement

**Table:**

Refinement on *F*^2^	Primary atom site location: structure-invariant direct methods
Least-squares matrix: full	Secondary atom site location: difference Fourier map
*R*[*F*^2^ > 2σ(*F*^2^)] = 0.042	Hydrogen site location: inferred from neighbouring sites
*wR*(*F*^2^) = 0.138	H-atom parameters constrained
*S* = 1.02	*w* = 1/[σ^2^(*F*_o_^2^) + (0.0791*P*)^2^ + 0.6172*P*] where *P* = (*F*_o_^2^ + 2*F*_c_^2^)/3
3757 reflections	(Δ/σ)_max_ = 0.006
226 parameters	Δρ_max_ = 0.18 e Å^−^^3^
0 restraints	Δρ_min_ = −0.26 e Å^−^^3^

### Special details

**Table:**

Geometry. All e.s.d.'s (except the e.s.d. in the dihedral angle between two l.s. planes) are estimated using the full covariance matrix. The cell e.s.d.'s are taken into account individually in the estimation of e.s.d.'s in distances, angles and torsion angles; correlations between e.s.d.'s in cell parameters are only used when they are defined by crystal symmetry. An approximate (isotropic) treatment of cell e.s.d.'s is used for estimating e.s.d.'s involving l.s. planes.
Refinement. Refinement of *F*^2^ against ALL reflections. The weighted *R*-factor *wR* and goodness of fit *S* are based on *F*^2^, conventional *R*-factors *R* are based on *F*, with *F* set to zero for negative *F*^2^. The threshold expression of *F*^2^ > σ(*F*^2^) is used only for calculating *R*-factors(gt) *etc*. and is not relevant to the choice of reflections for refinement. *R*-factors based on *F*^2^ are statistically about twice as large as those based on *F*, and *R*- factors based on ALL data will be even larger.

### Fractional atomic coordinates and isotropic or equivalent isotropic displacement parameters (Å^2^)

**Table:**

	*x*	*y*	*z*	*U*_iso_*/*U*_eq_	
S1	0.77988 (3)	1.09442 (6)	1.01400 (3)	0.0562 (2)	
C21	0.47261 (12)	1.2996 (2)	0.87474 (10)	0.0488 (5)	
H21	0.4282	1.3379	0.8657	0.059*	
C22	0.52896 (12)	1.3863 (2)	0.88829 (10)	0.0506 (5)	
H22	0.5226	1.4830	0.8876	0.061*	
C23	0.59526 (11)	1.33134 (19)	0.90300 (10)	0.0432 (4)	
H23	0.6334	1.3905	0.9118	0.052*	
C3	0.38780 (12)	0.6768 (2)	0.83907 (12)	0.0552 (6)	
H3	0.3499	0.6193	0.8271	0.066*	
C2	0.41860 (12)	0.7624 (3)	0.78966 (11)	0.0562 (6)	
H2	0.4012	0.7629	0.7441	0.067*	
C1	0.47491 (11)	0.8473 (2)	0.80711 (10)	0.0476 (5)	
H1	0.4941	0.9065	0.7734	0.057*	
C6	0.50367 (10)	0.84613 (18)	0.87452 (8)	0.0352 (4)	
C7	0.56618 (9)	0.94019 (17)	0.89155 (8)	0.0325 (4)	
C19	0.54721 (9)	1.09806 (17)	0.88904 (8)	0.0324 (4)	
C20	0.48144 (10)	1.1551 (2)	0.87441 (8)	0.0394 (4)	
H20	0.4433	1.0967	0.8644	0.047*	
C16	0.69820 (11)	1.0122 (2)	1.04320 (10)	0.0460 (5)	
H16A	0.7075	0.9194	1.0619	0.055*	
H16B	0.6757	1.0682	1.0792	0.055*	
C17	0.65229 (10)	1.00336 (17)	0.97976 (8)	0.0338 (4)	
C14	0.67205 (10)	1.10848 (17)	0.92219 (9)	0.0358 (4)	
C24	0.60395 (9)	1.18688 (18)	0.90446 (8)	0.0341 (4)	
C4	0.41364 (11)	0.6773 (2)	0.90609 (12)	0.0495 (5)	
H4	0.3923	0.6216	0.9400	0.059*	
C5	0.47154 (10)	0.76063 (19)	0.92374 (9)	0.0398 (4)	
H5	0.4889	0.7588	0.9693	0.048*	
C18	0.59965 (9)	0.91744 (17)	0.96419 (8)	0.0336 (4)	
H18	0.5840	0.8483	0.9948	0.040*	
C8	0.63048 (10)	0.92721 (17)	0.84115 (8)	0.0342 (4)	
C13	0.68638 (10)	1.01795 (18)	0.85762 (9)	0.0367 (4)	
C12	0.74681 (11)	1.0184 (2)	0.81727 (11)	0.0492 (5)	
H12	0.7839	1.0787	0.8282	0.059*	
C11	0.75247 (13)	0.9291 (2)	0.76038 (11)	0.0578 (6)	
H11	0.7929	0.9312	0.7326	0.069*	
C10	0.69867 (13)	0.8378 (2)	0.74485 (10)	0.0564 (6)	
H10	0.7032	0.7770	0.7071	0.068*	
C9	0.63726 (11)	0.8355 (2)	0.78527 (9)	0.0437 (5)	
H9	0.6010	0.7728	0.7748	0.052*	
C15	0.73314 (11)	1.1988 (2)	0.94933 (11)	0.0493 (5)	
H15A	0.7151	1.2841	0.9704	0.059*	
H15B	0.7647	1.2240	0.9113	0.059*	

### Atomic displacement parameters (Å^2^)

**Table:**

	*U*^11^	*U*^22^	*U*^33^	*U*^12^	*U*^13^	*U*^23^
S1	0.0443 (3)	0.0488 (3)	0.0754 (4)	0.0006 (2)	−0.0213 (3)	−0.0014 (2)
C21	0.0489 (12)	0.0560 (12)	0.0415 (10)	0.0226 (10)	−0.0013 (9)	0.0015 (8)
C22	0.0640 (14)	0.0391 (10)	0.0487 (11)	0.0181 (10)	−0.0023 (10)	0.0013 (8)
C23	0.0525 (12)	0.0339 (9)	0.0432 (10)	0.0031 (8)	−0.0014 (9)	0.0015 (7)
C3	0.0416 (12)	0.0508 (12)	0.0732 (14)	−0.0032 (9)	−0.0068 (10)	−0.0229 (11)
C2	0.0496 (13)	0.0706 (14)	0.0483 (11)	0.0040 (11)	−0.0110 (10)	−0.0191 (10)
C1	0.0459 (12)	0.0600 (12)	0.0370 (9)	0.0002 (10)	−0.0026 (8)	−0.0058 (8)
C6	0.0364 (10)	0.0358 (9)	0.0335 (8)	0.0028 (7)	0.0006 (7)	−0.0054 (7)
C7	0.0353 (9)	0.0361 (9)	0.0261 (8)	0.0015 (7)	0.0006 (6)	0.0000 (6)
C19	0.0368 (9)	0.0366 (9)	0.0237 (7)	0.0056 (7)	0.0029 (6)	0.0011 (6)
C20	0.0385 (10)	0.0484 (10)	0.0315 (8)	0.0065 (8)	0.0000 (7)	−0.0006 (7)
C16	0.0518 (12)	0.0429 (10)	0.0433 (10)	0.0047 (9)	−0.0123 (9)	−0.0045 (8)
C17	0.0386 (10)	0.0306 (8)	0.0321 (8)	0.0069 (7)	−0.0042 (7)	0.0003 (6)
C14	0.0341 (9)	0.0302 (8)	0.0430 (9)	0.0024 (7)	−0.0021 (7)	0.0024 (7)
C24	0.0371 (10)	0.0335 (9)	0.0316 (8)	0.0055 (7)	0.0007 (7)	0.0025 (6)
C4	0.0443 (12)	0.0400 (10)	0.0641 (13)	−0.0044 (9)	0.0030 (10)	−0.0043 (9)
C5	0.0415 (11)	0.0364 (9)	0.0415 (9)	−0.0004 (8)	−0.0001 (8)	−0.0038 (7)
C18	0.0392 (10)	0.0326 (9)	0.0290 (8)	0.0021 (7)	−0.0001 (7)	0.0023 (6)
C8	0.0381 (10)	0.0342 (9)	0.0303 (8)	0.0076 (7)	0.0021 (7)	0.0050 (7)
C13	0.0375 (10)	0.0344 (9)	0.0384 (9)	0.0078 (7)	0.0020 (7)	0.0090 (7)
C12	0.0396 (11)	0.0506 (12)	0.0576 (12)	0.0073 (9)	0.0084 (9)	0.0158 (9)
C11	0.0531 (14)	0.0686 (15)	0.0518 (12)	0.0231 (12)	0.0216 (10)	0.0156 (11)
C10	0.0692 (16)	0.0607 (13)	0.0394 (10)	0.0273 (12)	0.0119 (10)	−0.0001 (9)
C9	0.0518 (12)	0.0446 (10)	0.0349 (9)	0.0134 (9)	−0.0005 (8)	−0.0005 (7)
C15	0.0429 (12)	0.0375 (10)	0.0675 (13)	−0.0027 (8)	−0.0104 (10)	0.0032 (9)

### Geometric parameters (Å, º)

**Table:**

S1—C15	1.816 (2)	C16—C17	1.493 (2)
S1—C16	1.818 (2)	C16—H16A	0.9700
C21—C22	1.372 (3)	C16—H16B	0.9700
C21—C20	1.388 (3)	C17—C18	1.322 (2)
C21—H21	0.9300	C17—C14	1.534 (2)
C22—C23	1.386 (3)	C14—C24	1.526 (2)
C22—H22	0.9300	C14—C15	1.530 (3)
C23—C24	1.387 (2)	C14—C13	1.530 (2)
C23—H23	0.9300	C4—C5	1.393 (3)
C3—C4	1.371 (3)	C4—H4	0.9300
C3—C2	1.377 (3)	C5—H5	0.9300
C3—H3	0.9300	C18—H18	0.9300
C2—C1	1.377 (3)	C8—C9	1.386 (2)
C2—H2	0.9300	C8—C13	1.401 (3)
C1—C6	1.398 (2)	C13—C12	1.377 (3)
C1—H1	0.9300	C12—C11	1.385 (3)
C6—C5	1.385 (3)	C12—H12	0.9300
C6—C7	1.518 (2)	C11—C10	1.370 (3)
C7—C18	1.541 (2)	C11—H11	0.9300
C7—C19	1.548 (2)	C10—C9	1.394 (3)
C7—C8	1.555 (2)	C10—H10	0.9300
C19—C20	1.384 (2)	C9—H9	0.9300
C19—C24	1.397 (2)	C15—H15A	0.9700
C20—H20	0.9300	C15—H15B	0.9700
			
C15—S1—C16	91.89 (9)	C24—C14—C15	115.81 (14)
C22—C21—C20	120.39 (18)	C24—C14—C13	104.26 (14)
C22—C21—H21	119.8	C15—C14—C13	117.22 (16)
C20—C21—H21	119.8	C24—C14—C17	105.92 (14)
C21—C22—C23	120.73 (18)	C15—C14—C17	107.92 (15)
C21—C22—H22	119.6	C13—C14—C17	104.65 (13)
C23—C22—H22	119.6	C23—C24—C19	120.46 (17)
C24—C23—C22	119.09 (19)	C23—C24—C14	126.19 (16)
C24—C23—H23	120.5	C19—C24—C14	113.35 (14)
C22—C23—H23	120.5	C3—C4—C5	120.5 (2)
C4—C3—C2	119.25 (19)	C3—C4—H4	119.7
C4—C3—H3	120.4	C5—C4—H4	119.7
C2—C3—H3	120.4	C6—C5—C4	121.00 (18)
C3—C2—C1	120.55 (19)	C6—C5—H5	119.5
C3—C2—H2	119.7	C4—C5—H5	119.5
C1—C2—H2	119.7	C17—C18—C7	115.09 (15)
C2—C1—C6	121.21 (19)	C17—C18—H18	122.5
C2—C1—H1	119.4	C7—C18—H18	122.5
C6—C1—H1	119.4	C9—C8—C13	119.54 (17)
C5—C6—C1	117.42 (17)	C9—C8—C7	126.85 (17)
C5—C6—C7	122.88 (15)	C13—C8—C7	113.58 (14)
C1—C6—C7	119.66 (16)	C12—C13—C8	120.02 (17)
C6—C7—C18	115.41 (14)	C12—C13—C14	126.63 (18)
C6—C7—C19	112.82 (14)	C8—C13—C14	113.35 (15)
C18—C7—C19	105.06 (13)	C13—C12—C11	120.1 (2)
C6—C7—C8	115.31 (13)	C13—C12—H12	119.9
C18—C7—C8	103.10 (13)	C11—C12—H12	119.9
C19—C7—C8	103.83 (13)	C10—C11—C12	120.2 (2)
C20—C19—C24	119.52 (16)	C10—C11—H11	119.9
C20—C19—C7	126.56 (16)	C12—C11—H11	119.9
C24—C19—C7	113.91 (15)	C11—C10—C9	120.46 (19)
C19—C20—C21	119.77 (19)	C11—C10—H10	119.8
C19—C20—H20	120.1	C9—C10—H10	119.8
C21—C20—H20	120.1	C8—C9—C10	119.6 (2)
C17—C16—S1	105.52 (13)	C8—C9—H9	120.2
C17—C16—H16A	110.6	C10—C9—H9	120.2
S1—C16—H16A	110.6	C14—C15—S1	106.81 (13)
C17—C16—H16B	110.6	C14—C15—H15A	110.4
S1—C16—H16B	110.6	S1—C15—H15A	110.4
H16A—C16—H16B	108.8	C14—C15—H15B	110.4
C18—C17—C16	130.86 (16)	S1—C15—H15B	110.4
C18—C17—C14	115.23 (15)	H15A—C15—H15B	108.6
C16—C17—C14	113.86 (15)		
			
C20—C21—C22—C23	−1.1 (3)	C15—C14—C24—C19	−171.86 (15)
C21—C22—C23—C24	−0.5 (3)	C13—C14—C24—C19	57.81 (17)
C4—C3—C2—C1	0.2 (3)	C17—C14—C24—C19	−52.30 (18)
C3—C2—C1—C6	1.9 (3)	C2—C3—C4—C5	−1.6 (3)
C2—C1—C6—C5	−2.6 (3)	C1—C6—C5—C4	1.2 (3)
C2—C1—C6—C7	179.40 (18)	C7—C6—C5—C4	179.12 (17)
C5—C6—C7—C18	9.5 (2)	C3—C4—C5—C6	0.9 (3)
C1—C6—C7—C18	−172.62 (16)	C16—C17—C18—C7	−178.55 (17)
C5—C6—C7—C19	−111.32 (18)	C14—C17—C18—C7	−1.4 (2)
C1—C6—C7—C19	66.6 (2)	C6—C7—C18—C17	−177.47 (15)
C5—C6—C7—C8	129.62 (17)	C19—C7—C18—C17	−52.56 (19)
C1—C6—C7—C8	−52.5 (2)	C8—C7—C18—C17	55.91 (18)
C6—C7—C19—C20	1.0 (2)	C6—C7—C8—C9	−3.1 (2)
C18—C7—C19—C20	−125.52 (17)	C18—C7—C8—C9	123.63 (17)
C8—C7—C19—C20	126.54 (17)	C19—C7—C8—C9	−126.98 (17)
C6—C7—C19—C24	−179.93 (13)	C6—C7—C8—C13	178.59 (14)
C18—C7—C19—C24	53.54 (17)	C18—C7—C8—C13	−54.73 (17)
C8—C7—C19—C24	−54.39 (17)	C19—C7—C8—C13	54.66 (17)
C24—C19—C20—C21	0.4 (2)	C9—C8—C13—C12	1.7 (2)
C7—C19—C20—C21	179.44 (16)	C7—C8—C13—C12	−179.77 (15)
C22—C21—C20—C19	1.1 (3)	C9—C8—C13—C14	−177.77 (15)
C15—S1—C16—C17	32.38 (13)	C7—C8—C13—C14	0.72 (19)
S1—C16—C17—C18	155.10 (17)	C24—C14—C13—C12	123.19 (18)
S1—C16—C17—C14	−22.11 (18)	C15—C14—C13—C12	−6.3 (3)
C18—C17—C14—C24	55.15 (19)	C17—C14—C13—C12	−125.78 (18)
C16—C17—C14—C24	−127.18 (15)	C24—C14—C13—C8	−57.34 (17)
C18—C17—C14—C15	179.77 (16)	C15—C14—C13—C8	173.18 (15)
C16—C17—C14—C15	−2.6 (2)	C17—C14—C13—C8	53.70 (18)
C18—C17—C14—C13	−54.68 (19)	C8—C13—C12—C11	0.0 (3)
C16—C17—C14—C13	122.99 (16)	C14—C13—C12—C11	179.43 (17)
C22—C23—C24—C19	2.1 (3)	C13—C12—C11—C10	−1.4 (3)
C22—C23—C24—C14	−177.67 (17)	C12—C11—C10—C9	1.2 (3)
C20—C19—C24—C23	−2.0 (2)	C13—C8—C9—C10	−2.0 (3)
C7—C19—C24—C23	178.83 (15)	C7—C8—C9—C10	179.71 (16)
C20—C19—C24—C14	177.74 (14)	C11—C10—C9—C8	0.6 (3)
C7—C19—C24—C14	−1.39 (19)	C24—C14—C15—S1	144.72 (13)
C15—C14—C24—C23	7.9 (3)	C13—C14—C15—S1	−91.47 (17)
C13—C14—C24—C23	−122.42 (18)	C17—C14—C15—S1	26.26 (18)
C17—C14—C24—C23	127.46 (18)	C16—S1—C15—C14	−34.57 (15)

### Hydrogen-bond geometry (Å, º)

Cg1 and Cg2 are the centroids of the C19–C2 and C8–C13 rings, respectively.

**Table:**

*D*—H···*A*	*D*—H	H···*A*	*D*···*A*	*D*—H···*A*
C2—H2···*Cg*1^i^	0.93	2.80	3.516 (2)	135
C5—H5···*Cg*1^ii^	0.93	2.76	3.5844 (19)	149
C15—H15*B*···*Cg*2^iii^	0.97	2.98	3.845 (2)	149

Symmetry codes: (i) −*x*+3/2, −*y*−1, *z*+3/2; (ii) −*x*+1, −*y*+2, −*z*+2; (iii) *x*+1, −*y*−1/2, *z*−1/2.
